# Development of clinical pathways to improve multidisciplinary care of high-risk pediatric oncology patients

**DOI:** 10.3389/fonc.2022.1033993

**Published:** 2022-11-29

**Authors:** Agnes Reschke, Rebecca M. Richards, Stephanie M. Smith, Adrienne H. Long, Lianna J. Marks, Liora Schultz, Jennifer L. Kamens, Catherine Aftandilian, Kara L. Davis, Tanja Gruber, Kathleen M. Sakamoto

**Affiliations:** ^1^ Division of Hematology, Oncology, Stem Cell Transplantation and Regenerative Medicine, Department of Pediatrics, Stanford University School of Medicine, Stanford, CA, United States; ^2^ Division of Critical Care Medicine, Department of Pediatrics, Stanford University School of Medicine, Stanford, CA, United States; ^3^ Division of Pediatric Hematology, Oncology, and Bone Marrow Transplantation, University of Wisconsin-Madison, Madison, WI, United States; ^4^ Stanford Center for Cancer Cell Therapy, Stanford Cancer Institute, Stanford University, Stanford, CA, United States; ^5^ Department of Pediatrics, Stanford University School of Medicine, Stanford, CA, United States

**Keywords:** clinical pathways, pediatric oncology, high-risk patients, hyperleukocytosis, anterior mediastinal mass

## Abstract

Clinical pathways are evidence-based tools that have been integrated into many aspects of pediatric hospital medicine and have proven effective at reducing in-hospital complications from a variety of diseases. Adaptation of similar tools for specific, high-risk patient populations in pediatric oncology has been slower, in part due to patient complexities and variations in management strategies. There are few published studies of clinical pathways for pediatric oncology patients. Pediatric patients with a new diagnosis of leukemia or lymphoma often present with one or more “oncologic emergencies” that require urgent intervention and deliberate multidisciplinary care to prevent significant consequences. Here, we present two clinical pathways that have recently been developed using a multidisciplinary approach at a single institution, intended for the care of patients who present with hyperleukocytosis or an anterior mediastinal mass. These clinical care pathways have provided a critical framework for the immediate care of these patients who are often admitted to the pediatric intensive care unit for initial management. The goal of the pathways is to facilitate multidisciplinary collaborations, expedite diagnosis, and streamline timely treatment initiation. Standardizing the care of high-risk pediatric oncology patients will ultimately decrease morbidity and mortality associated with these diseases to increase the potential for excellent outcomes.

## Introduction

### The use of clinical pathways in pediatric oncology

Clinical pathways are evidence-based tools that feature a multidisciplinary approach to care, detail the steps involved in care, include timeframes or criteria-based progression, and aim to standardize care for a specific clinical problem (1). Their use in oncologic care may limit unwanted variation in clinical approach and improve quality, efficiency, and value of care (2, 3). In adult patients with cancer diagnoses, clinical pathways have been developed primarily for drug treatment regimens (3). There are fewer published pathways for pediatric oncology patients, but those that exist focus on managing complications of cancer treatment such as fever and neutropenia (F&N) (4, 5). Similar standardization and evidence-based coordination of care may be especially helpful for pediatric oncology patients who present with life-threatening complications of their new cancer diagnosis.

### A challenge in pediatrics - critically ill oncology patients

While disease-free survival for pediatric patients with a new diagnosis of leukemia or lymphoma has improved dramatically in the last 40 years (6), the outcomes for those who present with a life-threatening disease component remain disproportionately poor. A large retrospective multicenter analysis by Zinter et al. found that while pediatric cancer patients represent 4.2% of pediatric intensive care unit (PICU) admissions, they comprise 11.4% of PICU deaths and have an overall mortality of 6.8% (7). Other publications report an even higher mortality for pediatric cancer patients admitted to the ICU, ranging from 13% to 27.8% (8–10). Unfortunately, despite the overall improvement in oncologic outcomes in recent years, there has not been much progress in the outcomes of critically ill oncology patients. Two particularly high-risk populations include patients presenting with hyperleukocytosis secondary to leukemia, who have a mortality rate of up to 20% in the case of acute myeloid leukemia (11) and patients presenting with an anterior mediastinal mass, who have a 15% risk of respiratory or cardiovascular collapse (12). In these cases, arriving at a diagnosis with the least invasive approach and initiating cancer-directed treatment as quickly as possible are necessary to limit morbidity and mortality. As such, we identified these patient populations as candidates for standardization in management using a clinical pathway to expedite workup and treatment. At our institution, we have developed clinical pathways for the immediate triage and emergent management of two high-risk clinical conditions in pediatric oncology patients: hyperleukocytosis and anterior mediastinal masses.

## Hyperleukocytosis pathway

### Hyperleukocytosis, an oncologic emergency

Hyperleukocytosis, defined as a white blood cell (WBC) count greater than 100,000/µL, occurs in 10% to 20% of patients with newly diagnosed acute leukemia (11, 13, 14). While hyperleukocytosis is more common in acute lymphoblastic leukemia (ALL), acute myeloid leukemia (AML) is more likely to cause complications at lower relative WBC counts. The most concerning complication of hyperleukocytosis is leukostasis, a phenomenon that occurs when leukemic blasts occlude the microvasculature, obstructing blood flow to various tissues and leading to end organ damage. Leukemic blasts and endothelial cells interact on the molecular level to release tissue factors and proinflammatory cytokines that worsen tissue damage and lead to coagulopathy (15). Leukostasis may present with a variety of signs and symptoms, including acute kidney injury, oliguria, shortness of breath, hypoxemia, respiratory distress, altered mental status, visual changes, and focal neurologic deficits. Symptomatic leukostasis is a true medical emergency and requires prompt initiation of leukemia-directed therapy to reduce the risk of permanent organ damage or death. Complications related to leukostasis occur more often with AML due to the increased size and poor deformability of myeloblasts.

### Hyperleukocytosis pathway highlights

We took the clinical factors described above into account to create a clinical pathway with risk stratification based on the presence or absence of leukostasis, general clinical appearance, and suspected diagnosis from morphology. A mainstay of treatment for all patients with newly diagnosed leukemia – especially those with hyperleukocytosis – is hyperhydration to improve hyperviscosity and reduce the risk of leukostasis. For patients identified to be at low risk (no signs of leukostasis, well-appearing, and with morphology favoring ALL or chronic myeloid leukemia [CML]), leukemia-directed therapy is not initiated until confirmation of diagnosis by flow cytometry. However, symptomatic patients, those who are ill-appearing, or those with morphology favoring AML, acute promyelocytic leukemia (APML), or ambiguous morphology, warrant a more aggressive approach and initiation of immediate empiric therapy.

Of note, there is no definitive evidence demonstrating a mortality benefit for leukapheresis in patients with hyperleukocytosis and hence it is not a part of the treatment algorithm (14, 16). Leukapheresis may be considered in patients with AML with ongoing leukostasis as an adjunct to initiation of chemotherapy (11, 16–18) or patients with CML with evidence of priapism, hearing loss, visual changes, or pulmonary infiltrates (19). Initiation of leukapheresis is often time consuming as it requires placement of specialized central lines and mobilization of multiple services. As a result, our pathway stresses that leukapheresis should not delay initiation of definitive leukemia-directed therapy.

Other complications associated with hyperleukocytosis include tumor lysis syndrome (TLS) with related metabolic derangements and disseminated intravascular coagulation (DIC), which increases the risk of intracranial hemorrhage (ICH). While a separate clinical pathway guides the management of TLS at our institution, the hyperleukocytosis pathway details transfusion parameters, which are modified in patients with DIC and/or ICH. In these patients, the INR goal is lower and the platelet goal is higher to minimize the risk of bleeding.

**Figure 1:**
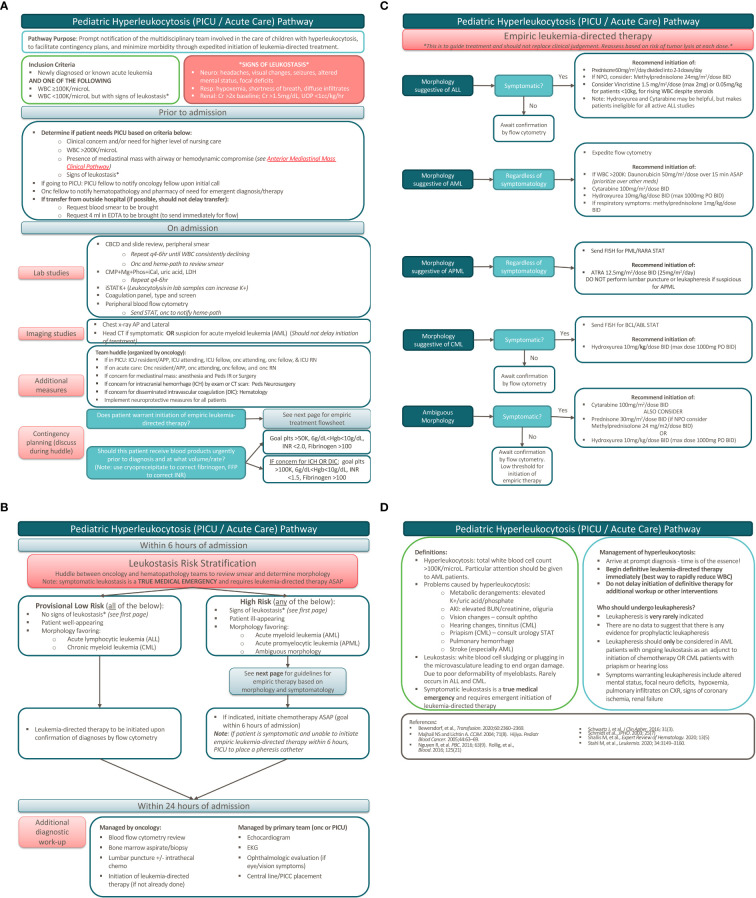
Pediatric hyperleukocytosis pathway. **(A)** Pathway overview. **(B)** Patient risk stratification. **(C)** Empiric leukemia-directed therapy. **(D)** Definitions and additional management.

Another important aspect of the management of hyperleukocytosis is surrounding these transfusion parameters. While these patients are often anemic at presentation, packed red blood cell (PRBC) transfusions in the setting of hyperleukocytosis can increase blood viscosity and result in worsening leukostasis. Therefore, PRBC transfusions should be reserved for patients who are hemodynamically unstable and should be done with extreme caution (20). In contrast, platelets have a minimal contribution to blood viscosity and given the risk of DIC and ICH, platelets should be transfused to maintain a platelet goal >50,000. If there is active concern for DIC or ICH, this threshold should be increased to maintain a plateletgoal >100,000. **Figure 1** depicts the most recent hyperleukocytosis pathway currently in use at Lucile Packard Children’s Hospital (LPCH), Stanford Children’s Health since October 2021.

### Hyperleukocytosis pathway development

This pathway was developed by a multidisciplinary group including physicians from both the Division of Pediatric Hematology, Oncology, Stem Cell Transplantation & Regenerative Medicine and the Division of Pediatric Critical Care, incorporating additional input from oncology pharmacists. This pathway underwent multiple iterations to incorporate feedback from members of both divisions. The goal of this pathway is prompt notification of the multidisciplinary team involved in the care of children with hyperleukocytosis to facilitate contingency planning and to minimize morbidity and mortality through the expedited initiation of leukemia-directed treatment.

## Anterior mediastinal mass pathway

### Anterior mediastinal mass, an oncologic emergency

Anterior mediastinal masses are another well-recognized oncologic emergency due to their precarious location that may result in narrowing or obstruction of the trachea, bronchi, superior vena cava, pulmonary vasculature, great vessels, and heart. Compression of the trachea or bronchi can present with dyspnea, orthopnea, cough, stridor, hoarseness, or chest pain and puts a patient at risk of airway obstruction and respiratory failure. A mass causing compression of the superior vena cava (also known as SVC syndrome) can cause a patient to present with edema of the head, neck, and upper extremities from increased venous pressure and decreased venous return, which can lead to thrombosis, cerebral edema, and hemodynamic instability. Compression of pulmonary arteries can result in decreased pulmonary perfusion and right heart failure, while compression of pulmonary veins can lead to pulmonary edema and decreased cardiac output. Compression of the heart and great vessels can result in decreased cardiac output and put a patient at high risk of cardiovascular collapse (20). In a patient with an anterior mediastinal mass, the degree of cardiorespiratory compromise can be evaluated with computed tomography (CT) and echocardiogram to further inform medical decision making.

### Anterior mediastinal mass pathway highlights

Similar to the hyperleukocytosis pathway, we incorporated clinical factors into a risk stratification schema to organize a multidisciplinary approach to determine the ideal diagnostic approach and treatment plan. Patients are considered “high-risk” if there is: 1) tracheal cross-sectional area ≤70% and/or presence of carinal/bronchial compression, 2) significant or near-complete SVC obstruction, 3) pericardial effusion and/or tamponade, 4) pulmonary artery outflow obstruction, or 5) ventricular dysfunction (21). In these cases, general anesthesia poses a significant risk to patients as it can decrease respiratory drive, cause loss of normal negative pressure ventilation, decrease bronchial smooth muscle tone increasing the risk of airway collapse, and cause peripheral vasodilation leading to decreased venous return (20). As such, recommendations include minimizing general anesthesia in favor of using local anesthesia whenever possible, and in conjunction with the oncologist, identifying the least invasive approach to diagnosis. When general anesthesia is necessary, it is important to maintain spontaneous ventilation, avoid muscle relaxants, and ensure the availability of additional equipment and extracorporeal membrane oxygenation (ECMO) in the case of emergency. Even with these precautions, the risk of life-threatening complications from anesthesia in these clinical scenarios has been reported to be as high as 9.4% to 20% (22).

The most common pediatric malignancies associated with anterior mediastinal masses include non-Hodgkin lymphoma (predominantly T-lymphoblastic leukemia/lymphoma) and Hodgkin lymphoma, with a smaller proportion being caused by germ cell tumors, thymomas, and thyroid tumors. Each malignancy is diagnosed differently, so the clinical picture needs to be considered in determining the study with the highest likelihood of yielding a diagnosis (23). While lymphoblastic leukemia or lymphoma can be diagnosed by flow cytometry if there are circulating malignant blasts, bone marrow biopsy can provide a diagnosis in 32% of patients with non-Hodgkin lymphoma and a mediastinal mass when peripheral disease is absent (23). Diagnostic yield of thoracentesis of pleural effusions can be as high as 92% in patients with lymphoblastic lymphoma, but is of limited utility for Hodgkin lymphoma. The most definitive approach regardless of diagnosis is biopsy of the primary site, which can be diagnostic in 86-100% of cases depending on the type of biopsy (23). The site of biopsy is another important consideration. In one study, only 19% of children required biopsy of the primary mass (24). A diagnostic algorithm, such as the one created by Perger et al, guides physicians to start with the least invasive approach and proceed to more invasive approaches if the prior approach was non-diagnostic. While this minimizes the initial procedural risk, it may lead to multiple procedures and a substantial delay in diagnosis. For this reason, we favor a multidisciplinary approach that harnesses each individual’s expertise to select a diagnostic strategy that is both high yield and minimally invasive, prioritizing obtaining a diagnostic specimen on the first attempt. Only with prompt diagnosis can definitive disease-directed therapy be initiated swiftly.

**Figure 2:**
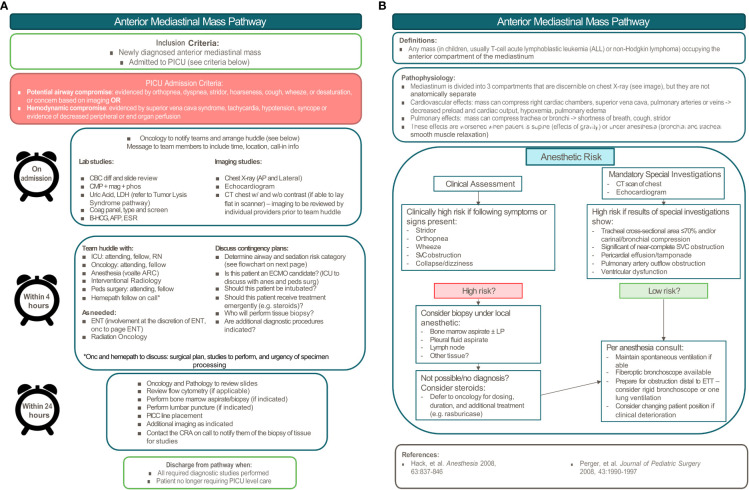
Pediatric anterior mediastinal mass pathway. **(A)** Pathway overview. **(B)** Anesthetic risk stratification.

The initial mediastinal mass treatment algorithm at our institution was created and published by Hammer in 2004 (25). This pathway has been revised multiple times since then and has incorporated recommendations for anesthetic risk stratification from other studies (21). While 3-level risk stratification strategies have more recently been developed for the management of children with anterior mediastinal masses (22, 26), the approach to intermediate and high-risk patients remains the same, advocating for the avoidance of general anesthesia in both groups when possible. The most recent iteration of the LPCH pathway includes a timeline to expedite a multidisciplinary huddle and determine the diagnostic/treatment approach. **Figure 2** depicts the anterior mediastinal mass pathway currently in use at LPCH, Stanford Children’s Health since March 2020.

### Anterior mediastinal mass pathway development

While the original anterior mediastinal mass pathway at LPCH was developed by Hammer (25), a pediatric intensivist and anesthesiologist, it has since been revised by a multidisciplinary group including physicians from the Divisions of Pediatric Hematology, Oncology, Stem Cell Transplantation & Regenerative Medicine, the Division of Critical Care, the Division of Pediatric Anesthesiology, the Division of Pediatric Surgery, the Division of Pediatric Interventional Radiology, and the Division of Pediatric Radiology. It was presented at the Surgical and Procedural Quality Committee Meeting with members of all subspecialties present and approved for implementation. The goals of this pathway are to: 1) standardize a systematic approach to pediatric patients with an anterior mediastinal mass, 2) enhance communication between various teams, 3) minimize risk to patients and improve patient safety, and 4) expedite diagnosis to initiate treatment as soon as possible.

## Pathway implementation

Both pathways are published on the Stanford Children’s Health Intranet Clinical Pathways website. They are accessible to all who work at LPCH through a link directly accessible through the electronic health record. Information about the implementation of these pathways was disseminated to all fellows and physicians in the Divisions of Pediatric Hematology, Oncology, Stem Cell Transplantation & Regenerative Medicine and Pediatric Critical Care through joint educational conferences.

## Discussion

The risk of mortality for pediatric patients presenting with hyperleukocytosis or a mediastinal mass is well-established. Minimizing time to diagnosis and initiation of treatment is key to improving outcomes. The primary goal of both clinical pathways presented here is to standardize and expedite management for critically ill pediatric oncology patients at diagnosis to decrease morbidity and mortality. Patients who present with hyperleukocytosis or a mediastinal mass are ideal candidates for this type of emergent, focused, multidisciplinary care. These pathways, created by individuals in both the Division of Pediatric Hematology, Oncology, Stem Cell Transplantation & Regenerative Medicine and the Division of Pediatric Critical Care, facilitate collaboration and ensure that all teams are working within a well-defined roadmap on a concrete timeline to achieve these goals. Another important element of these pathways is the multidisciplinary discussion surrounding contingency planning. By anticipating questions that may arise (i.e. transfusion parameters) and discussing them as a team beforehand, we ensure that management decisions limit the patient’s risk of an adverse outcome. One especially important point of discussion to occur with patients presenting with an anterior mediastinal mass is the question of ECMO candidacy, and should a patient be deemed high-risk and an ECMO candidate, then plans can be made for the ECMO team to be on standby.

As tissue diagnosis can be obtained from peripheral blood flow cytometry in the vast majority of patients with hyperleukocytosis, this pathway advocates for the initiation of treatment in all high-risk patients without performing either a bone marrow evaluation or a lumbar puncture unless it is absolutely necessary. Awaiting completion of these procedures can lengthen the time to initiation of definitive treatment, further increasing a patient’s risk of leukostasis and end organ damage. In contrast, for low-risk patients, we often obtain both the bone marrow aspirate/biopsy and lumbar puncture prior to initiation of treatment as is standard of care.

The focus on obtaining diagnostic tissue prior to initiation of treatment is an important paradigm for treating patients who present with a mediastinal mass. Empiric therapy can result in a delay in diagnosis or rarely, inability to obtain a diagnosis, as these tumors are often very steroid and chemo-responsive. In some cases, empiric treatment may upstage a patient due to pre-treatment status, which could result in more aggressive therapy and thus may increase the risk of treatment-related adverse effects. Empiric therapy before obtaining a diagnostic specimen should be reserved as a lifesaving measure in patients in or at high-risk of respiratory or cardiovascular failure in whom diagnostic tissue cannot be safely obtained.

A large focus of many pediatric oncology practices around the country is to provide patients with the opportunity to enroll in therapeutic clinical trials as data has demonstrated improved outcomes when enrolled (27). In developing the treatment algorithm for empiric leukemia-directed therapy in patients with hyperleukocytosis, careful consideration was given to choosing chemotherapeutic agents that would be effective and would not preclude a patient’s ability to enroll in a clinical trial whenever possible.

While multidisciplinary approaches to the management of pediatric patients with anterior mediastinal masses have been described in the literature (24) and anterior mediastinal mass pathways have been created and implemented at other institutions (21, 23, 28), this pathway provides more comprehensive and directed guidance including a suggested timeline, contingency planning, and risk stratification. This is also the first published pediatric hyperleukocytosis clinical pathway and likewise provides a thorough and comprehensive approach to the management of these patients. This pathway also highlights a novel approach to these patients wherein the urgency of initiation of treatment is determined by presence of or estimated risk of leukostasis. Initiation of empiric therapy is often essential for high risk patients, though there are no current consensus guidelines for which agents (or the specific dosing) to select. During the creation of this pathway, we developed recommendations for empiric leukemia-directed therapy based on expertise and consensus within the faculty and pharmacists of the LPCH Division of Hematology, Oncology, Stem Cell Transplantation & Regenerative Medicine.

The development and successful implementation of hyperleukocytosis and anterior mediastinal mass pathways demonstrates the feasibility of standardizing the approach to these patients. While some aspects of these pathways may be controversial and may not be achievable at all institutions, we hope pediatric institutions can use these as a framework to develop their own clinical pathways to further improve outcomes for these vulnerable patient populations.

## Future Directions

Through the standardization and expedition of management for these uniquely at-risk pediatric oncology patients, we hope to improve patient outcomes. An ongoing effort at our institution is to provide practical implementation tools including the development of hyperleukocytosis and anterior mediastinal mass order sets in Epic that are directly linked to this pathway. In addition, we are currently developing hyperleukocytosis chemotherapy roadmaps and electronic treatment plans to expedite delivery of chemotherapy to the patient to facilitate treatment. We anticipate that these efforts will further streamline the care of these patients and improve time to treatment.

The anterior mediastinal mass and hyperleukocytosis pathways were launched in March 2020 and October 2021, respectively. After both pathways have been live for three years, we will conduct a single institution retrospective review to measure time to administration of chemotherapy and/or steroids prior to and after the implementation of each of these pathways. Secondary outcomes will include morbidity, mortality, and PICU length of stay. We anticipate improvement of outcomes with full implementation of these pathways, and we envision collaborative efforts with existing pediatric oncology-PICU networks to continually improve standards of care for critically ill pediatric oncology patients.

## Data availability statement

The original contributions presented in the study are included in the article/supplementary material. Further inquiries can be directed to the corresponding author.

## Author contributions

All of the authors contributed to writing, editing, and organization of the manuscript. All authors contributed to the article and approved the submitted version.

## Acknowledgments

The authors thank Michael Link, MD, Kate Steffen, MD, Felice Su, MD, Norman Lacayo, MD, Sean Green, PharmD, Shellie Josephs, MD, Kimberly Hazard, MD, Dita Gratzinger, MD, James Dunn, MD, Bill Chiu, MD, Gregory Hammer, MD, and James Fehr, MD at Lucile Packard Children’s Hospital, Stanford for their contributions to the development of these pathways.

## Conflict of interest

The authors declare that the research was conducted in the absence of any commercial or financial relationships that could be construed as a potential conflict of interest.

## Publisher’s note

All claims expressed in this article are solely those of the authors and do not necessarily represent those of their affiliated organizations, or those of the publisher, the editors and the reviewers. Any product that may be evaluated in this article, or claim that may be made by its manufacturer, is not guaranteed or endorsed by the publisher.
